# Supplementary material for: Earlier Detection of Alzheimer’s Disease Based on a Novel Biomarker cis P-tau by a Label-Free Electrochemical Immunosensor
Source: Biosensors (Basel). 2022 Oct 17;12(10):879. doi: 10.3390/bios12100879 (PMC9599477; doi:10.3390/bios12100879)
Supplement: Supplementary file 1 [file biosensors-12-00879-s001.zip › biosensors-1941374-supplementary.pdf]

biosensors

MDPI

Supplementary data

Early Detection of Alzheimer's disease using a biomarker *cis* p-tau by a novel label-free electrochemical immunosensor

Ayoub Shiravandi [1], Farzaneh Yari [2], Nahid Tofigh [3], Mohammad Kazemi Ashtiani [1], Koorosh Shahpasand [4], Mohammad-Hossein Ghanian [1,*], Faezeh Shekari [5,*] and Farnoush Faridbod [2,*]

[1] Department of Cell Engineering, Cell Science Research Center, Royan Institute for Stem Cell Biology and Technology, ACECR, Tehran, Iran
[2] Center of Excellence in Electrochemistry, School of Chemistry, College of Science, University of Tehran, Tehran, Iran
[3] Laboratory of Neuro-Organic Chemistry, Institute of Biochemistry and Biophysics (IBB), University of Tehran, Tehran, Iran
[4] Department of Stem Cells and Developmental Biology, Cell Science Research Center, Royan Institute for Stem Cell Biology and Technology, ACECR, Tehran, Iran
[5] Advanced Therapy Medicinal Product Technology Development Center, Royan Institute for Stem Cell Biology and Technology, ACECR, Tehran, Iran
* Correspondence: faezehshekari@gmail.com

1. Optimization of working conditions

To determine the optimal pH of medium for maximal electrochemical signal production in response to *cis* p-tau, DPV was performed using electrolytes with different values of pH varying from 6 to 9 and the difference between the measured currents at each pH was calculated as an indication of the performance of the immunosensor (Figure S1). The maximum current changes were observed at neutral pH (7.0 and 7.5) and at higher and lower values of pH, the current changes in the two states (with and without *cis* p-tau) were remarkably reduced, which can be attributed to the weaker interaction of *cis* p-tau with its antibody under basic or acidic conditions, probably due to structural changes, protonation or deprotonation, in *cis* p-tau or anti-*cis* p-tau mAb. According to the results, the pH of 7.5 was chosen as the optimum condition for all subsequent experiments.

The signal-to-noise ratio (SNR) was also studied as an indication of higher signal strength to noise and data reliability. The SNR was measured from the ratio of change in current after analyte capture to the change in baseline current due to intrinsic fluctuations of the immunosensor [67]. Five similar immunosensors were analyzed by DPV method and SNR was calculated by $SNR=I_s/I_n$, where I_s is the diagnostic signal and I_n is the amount of immunosensor noise current [64]. The SNR was obtained ≥3 for all the samples.

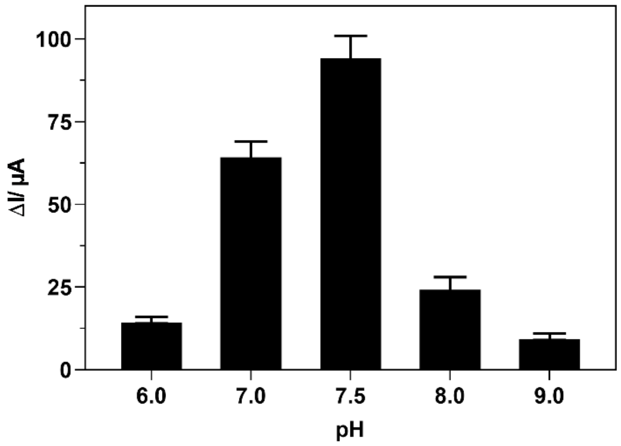

Figure S1. Response characterization of immunosensor to *cis* p-tau in PBS (1.0×10^{-12} M) at different pH values of the solution containing the 10 mM $K_4Fe(CN)_6/K_3Fe(CN)_6$ (1:1 ratio), analyzed by DPV method (n=3).

2. Selectivity of immunosensor

With the begin of the brain symptoms of Alzheimer's disease, the *trans* p-tau isomer also increases, like *cis* p-tau. Hence, a concern is the possibility of non-specific interaction with the *trans* p-tau isomer which can produce a false-positive response. To evaluate the possibility of an off-target response to *trans* p-tau, the DPV responses of the immunosensor to *trans* p-tau with high concentrations in PBS (100, 1000, and 2000 pM) were studied (Figure S2). The results showed that the presence of *trans* p-tau, even at much higher values than its normal values, cannot significantly change the signal produced with the blank PBS. Hence *trans* p-tau interference is not concerning and the current changes in response to real samples can be related directly to the *cis* p-tau concentration.

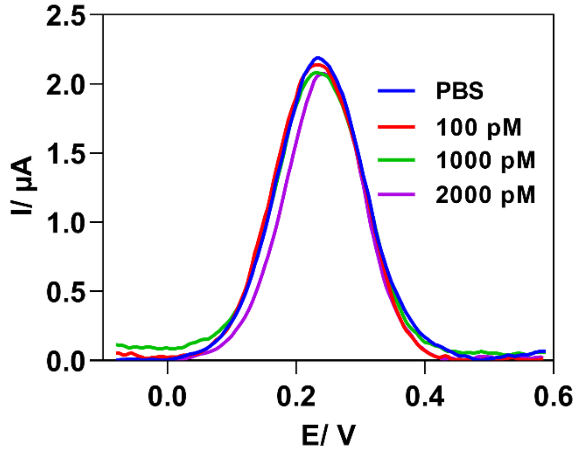

Figure S2. The DPV response of immunosensor to different concentrations of *trans* p-tau in 0.1 M PBS containing 10 mM of $[Fe(CN)_6]^{3-/4-}$ as a redox probe.

2. Immunosensor response to human CSF samples spiked with *cis* p-tau

Using the standard addition method, *cis* p-tau retrieval can be checked in the presence of the disturbing species in the complex matrix of real sample. In this method, the standard (*cis* p-tau) was added at known small amounts to the real samples and then the new concentration was measured based on the produced electrochemical response to study the ability of immunosensor for recovery of the added standard. To this end, the

CSF samples collected from healthy or diseased human subjects were spiked with different concentrations of *cis* p-tau standard and analyzed by DPV (Table S1). The added standards were recovered with high accuracy (97.9-106.3% recovery) and repeatability (RSD<5.33%). The sensitivity of immunosensor in response to *cis* p-tau additions in CSF was similar to that seen for *cis* p-tau in PBS, as the current intensity was linearly declined with increasing *cis* p-tau concentration over the whole of the required working range (Figure S3).

Table S1. Precision (RSD%) and recovery study of the immunosensor performed by adding standard solutions of *cis* p-tau to human CSF samples obtained from healthy and Alzheimer's individuals at different stages of AD (n=3).

Sample	Added *cis* p-tau (pM)	Found *cis* p-tau (pM)	Measured *cis* p-tau (pM)	RSD (%)	Recovery (%)
Healthy	0	0	15 ± 0.8	5.33	-
	20	21 ± 1.63	36 ± 0.8	2.26	102.8
	40	41.6 ± 0.47	56.6 ± 0.94	1.66	102.9
	60	61.6 ± 0.47	76.6 ± 1.24	1.62	102.1
	80	83 ± 1.41	98 ± 1.63	1.66	103.2
MCI	0	0	59 ± 2.16	3.66	-
	20	25 ± 1.41	84 ± 2.94	3.5	106.3
	40	40.6 ± 4.1	99.6 ± 2	2	100.6
	60	62.3 ± 3.3	121.3 ± 1.7	1.4	101.9
	80	82.3 ± 3.77	141.3 ± 2.85	2.2	101.6
Dementia	0	0	82.3 ± 1.24	1.5	-
	20	20 ± 3.26	102.3 ± 2	2	100
	40	39.3 ± 3.85	121.6 ± 2.5	2.05	99.4
	60	57 ± 2.94	139.3 ± 1.7	1.22	97.9
	80	79.7 ± 1.24	162 ± 2.16	1.33	99.8

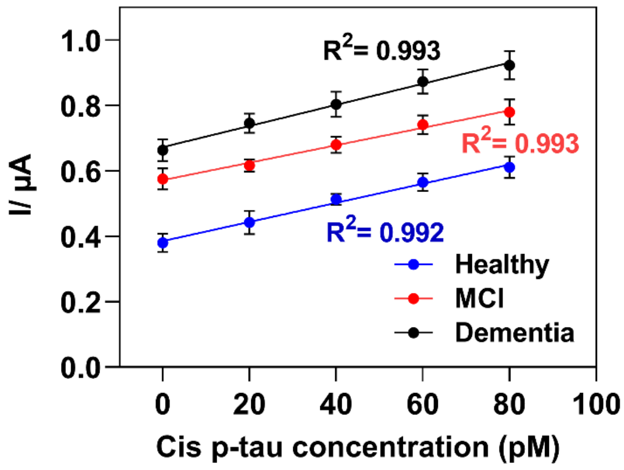

Figure S3. DPV response of the immunosensor to added standard solutions of *cis* p-tau to human CSF samples obtained from healthy and Alzheimer's individuals at different stages of AD (n=3).

3. Stability of immunosensor

To investigate the stability of immunosensor during long-term storage at 4 °C, DPV responses to *cis* p-tau in PBS (10 pM) were recorded over a three-month period with no chemical pretreatment (Figure S4A). The results showed that signal intensity was attenuated slightly from the beginning to the end of the period, so that the current produced on day 90 decreased by only 3.5% compared to the first day (Figure S4B). These data suggests that the immunosensor can maintain its function in an acceptable level required for detection after long-term storage.

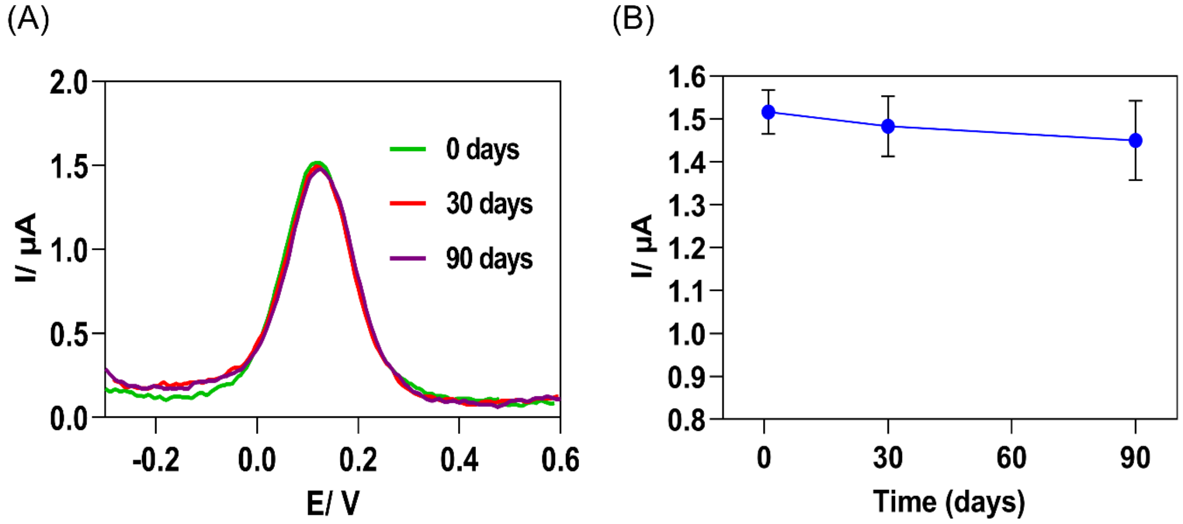

Figure S4. Comparison of immunosensor DPV response to *cis* p-tau in PBS (10×10^{-12} M) after immunosensor storage at 4 °C over a 90 days period (n=9).
